# Methylphenidate Multiphasic Release Tablet: Bioequivalence Assessment between Two Formulations Administered under Fasting and Fed Conditions

**DOI:** 10.3390/pharmaceutics15061737

**Published:** 2023-06-14

**Authors:** Marcelo Gomes Davanço, Jessica Meulman, Thalita Martins da Silva, Fernando Costa, Karini Bruno Bellorio, Iram Moreira Mundim, Ana Carolina Costa Sampaio, Leonardo de Souza Teixeira, Celso Francisco Pimentel Vespasiano

**Affiliations:** 1 Medical Department, Adium S.A., São Paulo 04794-000, SP, Brazil; jessica.meulman@adium.com.br (J.M.); thalita.silva@adium.com.br (T.M.d.S.); celso.vespasiano@adium.com.br (C.F.P.V.); 2 Medical Department, Monte Verde S.A., Munro, Buenos Aires B1605EBQ, Argentina; fcosta@raffo.com.ar; 3 Bioequivalence Unit, Instituto de Ciências Farmacêuticas de Estudos e Pesquisas, Goiânia 74175-100, GO, Brazil; karini.bellorio@icf.com.br (K.B.B.); iram.mundim@icf.com.br (I.M.M.); ana.cacula@icf.com.br (A.C.C.S.); leonardo.teixeira@icf.com.br (L.d.S.T.)

**Keywords:** methylphenidate, attention deficit/hyperactivity disorder (ADHD), bioequivalence, pharmacokinetics, bioavailability, extended-release

## Abstract

Methylphenidate hydrochloride is used to treat children, adolescents, and adults with attention deficit/hyperactivity disorder (ADHD). Multiphasic release formulation has been used to control drug levels, mainly during children’s school period. This study aimed to evaluate the bioequivalence between two methylphenidate hydrochloride extended-release tablets to meet regulatory requirements for registration in Brazil. Two independent studies (under fasting and fed conditions) designed as open-label, randomized, single-dose, two-period, two-way crossover trials were conducted in healthy subjects of both genders. Subjects were enrolled and randomly received a single dose of the test formulation methylphenidate hydrochloride 54 mg extended-release tablet (Consiv^®^, Adium S.A., São Paulo, Brazil) or the reference formulation (Concerta^®^, Janssen-Cilag Farmacêutica Ltd., São Paulo, Brazil), in each period, with a 7-day washout interval. Serial blood samples were collected up to 24 h post dose and methylphenidate plasma concentrations were obtained using a validated LC-MS/MS method. A total of 96 healthy subjects were enrolled in the fasting study, of which 80 completed the study. For the fed study, 52 healthy subjects were enrolled, and 46 subjects completed it. In both studies, 90% confidence intervals for Cmax, AUC_0–t_, AUC_0–inf_, and partial AUCs were within the acceptable limits of 80.00 to 125.00%. Thus, according to regulatory requirements, the test formulation (Consiv^®^) was considered to be bioequivalent to the reference formulation (Concerta^®^) in both conditions (fasting and fed) and, therefore, it can be considered interchangeable in clinical practice. Both formulations were safe and well tolerated in single-dose administration.

## 1. Introduction

Attention deficit/hyperactivity disorder (ADHD) is characterized as a persistent pattern of inattention or hyperactivity and is considered to be the most common neurodevelopment disorder [1]. Considering the latest official population figures in Brazil (2010) [2], ADHD reaches between 5.3% of youths and 2.5% of adults [3,4]. If untreated, ADHD could negatively affect the general patient’s quality of life; social, family, and professional relationships; and increase accidental injury, substance abuse, or other psychiatric morbidity risks [5]. Among the available treatments, central nervous system stimulants such as methylphenidate (MPH) and amphetamines are the most effective, in combination with psychological intervention. The indications include the treatment of children over six years of age, adolescents, and adults with significant impairment due to ADHD [6].

Early ADHD treatment was initially quite limited by available formulations. The immediate-release formulations required the use of multiple daily doses, which contributed to treatment adherence problems for school-age children, as well as associated safety concerns and high variability individual response. Over the past 15 years, there has been a significant expansion in the number of formulations available to ADHD patients, in hopes of better adherence and better long-term outcomes. Some of the main advantages of ER formulations for school-age children are the better pharmacokinetic (PK) profile (lower peak concentrations) and the ability to provide coverage throughout the day, which avoids the need to administer doses during school hours [7,8].

Currently, there are MPH formulations commercialized as multiphasic drug release systems. They were designed to release an initial amount of the drug immediately after administration, followed by slower drug delivery throughout the day [6]. Once administered in the morning, the early peak concentration is needed to control morning hyperactivity, while the later peak is needed to control hyperactivity during school hours. Thus, the ideal scenario is for plasma levels to decrease 10 h after administration, so that the stimulation is minimal, avoiding cases of insomnia. The presence of multiple peaks of maximum concentration is directly related to the efficacy of the drug but makes it difficult to establish bioequivalence [9]. According to the Concerta^®^ label, the MPH extended-release formulation has an action onset of 1 to 2 h after administration. The plasma concentrations continue to gradually increase, maintaining the clinical effect until 12 h. The Tmax is typically in the range of 6 to 8 h and the drug could be administered with or without food, with no bioavailability impact [6]. Nonetheless, a delay in MPH absorption is observed when it is taken with high-fat meals, which could alter the clinical response profile [10].

Generic medicines are considered to be therapeutically equivalent to the corresponding reference products if they have the same efficacy and safety and are, therefore, interchangeable. The comparison between test and reference formulations is performed through pharmacokinetic profiles and bioequivalence assessment to guarantee that both drug formulations present the same rate and extent of absorption [11,12].

For the design of bioequivalence studies with MPH extended-release tablets, the FDA Product Specific Guidance recommends the use of the partial area under the curve (pAUC) metrics in bioequivalence assessment, in addition to the traditional primary pharmacokinetic parameters (Cmax and AUC_0–t_). These metrics relate the drug’s release profile to its pharmacodynamic (PD) properties [13,14]. The PK/PD relationships of MPH were elucidated by Swanson (1978), comparing the clinical response time to drug plasma concentration, showing clinical superiority to the formulation with the higher concentration [15]. Therefore, pAUC metrics seem fundamental in developing generic and branded-generic formulations of MPH extended-release tablets.

The present study aimed to assess the bioequivalence and tolerability of two formulations of MPH 54 mg extended-release tablets administered under fasting and fed conditions to attend regulatory requirements for generic drug product registration in Brazil [11,12].

## 2. Materials and Methods

### 2.1. Ethical Aspects and Good Clinical Practices

Both fasting and fed study protocols were approved by the Research Ethics Committee of the Instituto de Ciências Farmacêuticas de Estudos e Pesquisas (Aparecida de Goiânia, Goiás, Brazil) with protocol numbers 4,108,995 and 3,548,871, respectively. The clinical, analytical, and statistical phases were performed at the Instituto de Ciências Farmacêuticas de Estudos e Pesquisas (Goiânia, Goiás, Brazil), a Brazilian clinical research center certified by ANVISA to conduct bioequivalence studies.

Both studies were conducted in compliance with the ethical principles of the Good Clinical Practices Guidelines [16], the Declaration of Helsinki [17], the local laws [18], and requirements for bioequivalence studies [11,12]. All subjects gave their informed consent for inclusion before the initiation of study procedures.

### 2.2. Study Design and Subjects

Two independent studies were performed, one under fasting and the other under fed conditions. Both studies were conducted as a single-center, open-label, randomized, balanced, single-dose, 2-period, 2-treatment, and 2-sequence crossover design.

Ninety-six (96) adult healthy subjects of both genders (48 male and 48 nonpregnant female subjects) were enrolled in the fasting study. Fifty-two (52) adult healthy subjects of both genders (26 male and 26 nonpregnant female subjects) were enrolled in the fed study. The sample size for both studies was calculated considering a within-subject variability (CV_ws_ = 30%) obtained from pilot studies performed by the sponsor (data unpublished).

Regarding the inclusion and exclusion criteria, for both studies, subjects had not previously participated in another clinical trial nor donated blood during the preceding six months and had no history of alcohol or drug abuse. They were aged between 18 and 50 years with a body mass index (BMI) between 18.5 and 28.6 kg/m^2^.

All subjects showed good health conditions or the absence of significant diseases after assessing their medical history, verifying vital signs, and conducting physical examinations, electrocardiograms, and routine laboratory tests. In addition, subjects must not have had lactose intolerance, a positive or indeterminate result for the RT-qPCR test for SARS-CoV-2, be smokers, be vegetarians, or have dietary habits that would prevent the intake of the provided fed study diet. They also should not have a clinical history or episodes of gastrointestinal disorders or have taken medications that would interfere with the pharmacokinetics of methylphenidate.

### 2.3. Formulations Studied

The test formulation was Consiv^®^, a methylphenidate hydrochloride 54 mg extended-release tablet (batch No. 84497, expiry date: February 2021) manufactured by Monte Verde S.A. (San Juan, Argentina) and imported to Brazil by Adium S.A. (Pindamonhangaba, São Paulo, Brazil). The reference formulation was Concerta^®^ 54 mg extended-release tablet (batch No. 8LE798, expiry date: September 2020) manufactured by Janssen Cilag Manufacturing LLC (Rockford, IL, USA) and imported to Brazil by Janssen-Cilag Farmacêutica Ltd. (São Paulo, Brazil). The same products and batches were administered in both the fasting and fed studies.

### 2.4. Drug Administration and Sampling Times

Fasting study: Each period began with a minimum overnight fasting period of eight hours. The subjects received a single dose of 54 mg MPH extended-release tablets from one of the two formulations, along with 200 mL of water. Following drug administration, the subjects remained fasted for four hours, with restrictions on water intake from seven hours before to two hours after drug administration. The diet, including food and drink, was standardized for all subjects in both periods. Alcoholic beverages, as well as food or beverages containing caffeine or xanthine (such as coffee, tea, chocolate, and cola- or guarana-based soft drinks), were not allowed in the 24 h before study admission. A total of 25 blood samples were collected at 0 h (before drug administration) and 0.33, 0.67, 1.00, 1.33, 1.67, 2.00, 2.50, 3.00, 3.50, 4.00, 4.50, 5.00, 5.50, 6.00, 6.50, 7.00, 7.50, 8.00, 8.50, 9.00, 10.0, 12.0, 16.0, and 24.0 h after drug administration, in tubes containing K_3_EDTA as the anticoagulant.

Fed study: In each period, the subjects were given a hypercaloric meal consisting of approximately 1000 kcal, 30 min before drug administration. Then, they receive a single dose of 54 mg of MPH extended-release tablets from one of the two formulations along with 200 mL of water. Following drug administration, the subjects remained fasted for four hours, with restrictions on water intake from seven hours before to two hours after drug administration. The diet, including food and drink, was standardized for all subjects in both periods, to maintain the standardization of treatment groups. Alcoholic beverages, and food or beverages containing caffeine or xanthine (such as coffee, tea, chocolate, and cola- or guarana-based soft drinks), were not allowed in the 24 h before study admission. A total of 26 blood samples were collected at 0 h (before drug administration) and 0.33, 0.67, 1.00, 1.50, 2.00, 2.50, 3.00, 3.50, 4.00, 4.50, 5.00, 5.50, 6.00, 6.50, 7.00, 7.50, 8.00, 8.50, 9.00, 9.50, 10.0, 11.0, 12.0, 16.0, and 24.0 h after drug administration, in tubes containing K_3_EDTA as the anticoagulant.

The blood samples from both studies were centrifuged at 3000 rpm for 5 min at 4 °C; the plasma was separated (2 mL) and transferred into cryogenic tubes. Then, 100 µL of citric acid 10% was immediately added to 2 mL of plasma and homogenized. Finally, the samples were stored at −80 °C with appropriate labeling until sample analysis.

### 2.5. Bioanalytical Method

The plasma samples were analyzed using validated high-performance liquid chromatography-tandem mass spectrometry (HPLC-MS/MS), to obtain MPH concentrations. The system includes an Agilent 1200 Series (Agilent Technologies Inc., Santa Clara, CA, USA) and an API 5000 MS/MS (Applied Biosystems/Sciex, Framingham, MA, USA). The analytes were extracted from the plasma using a protein precipitation method and methylphenidate-d9 was used as an internal standard (IS). To avoid interassay variations, all samples from the same participant were assessed in the same analytical run.

An amount of 3.0 µL of each sample was injected onto a Zorbax Eclipse XDB-Phenyl (4.6 × 150 mm; 3.5 µm) column, maintained at 20 °C. The mobile phase for MPH consisted of a mixture (70:30) of (A) acetonitrile and (B) ammonium acetate 5 mM solution (*v*/*v*, with 0.025% formic acid). The flow rate was 1 mL/min, in an isocratic performance. The detection of MPH was carried out in the mass spectrometer with the positive electrospray ionization multiple-reaction monitoring mode set to transmit at m/z 234.1 → 84.1 for MPH and m/z 243.3 → 93.2 for methylphenidate-d_9_ (IS).

The analyte concentrations were calculated through interpolation on the calibration curve, and the linearity range used was from 25 to 30,000 pg/mL. The bioanalytical method was validated in compliance with ANVISA guidance for bioanalytical method validation [19] including the evaluation of selectivity, concomitant medication interference, matrix effect, carry-over, calibration curve, precision, accuracy, reinjection reproducibility, and the stabilities of MPH under different conditions.

### 2.6. Pharmacokinetic and Statistical Analysis

The pharmacokinetic parameters were obtained from the curves of MPH plasma concentration versus time and statistically compared for the determination of bioequivalence in both the fasting and fed studies using Phoenix WinNonlin™ version 6.4 (Princeton, NJ, USA). The calculation of the area under the curve from zero to the last quantifiable concentration (AUC_0–t_) was performed using the trapezoidal method, and the area under the curve from zero to infinity (AUC_0–inf_) was calculated using the formula AUC_0–t_ + (Cn/kel), where Cn was the last quantifiable plasma concentration. The elimination constant (kel) was determined by analyzing the elimination phase of the graph depicting the log plasma concentration versus time. t_1/2_ was defined using the equation t_1/2_ = Ln(2)/kel and the maximum plasma drug concentration (Cmax) was obtained directly from the experimental data, as well as the time of the occurrence of Cmax (Tmax).

The Concerta^®^ tablet is an extended-release formulation with a bimodal release profile (designed to release a bolus of MPH followed by slower delivery later in the day) [6]. Thus, as per FDA product-specific guidance for generic drug development of MPH, the following three partial AUC (pAUC) metrics were necessary in addition to the traditional (Cmax and AUC_0–t_) metrics for each study:

- Fasting Study: log-transformed AUC_0–3_, AUC_3–7_, and AUC_7–12_, where AUC_0–3_ is the area under the plasma concentration vs. time curve from 0 to 3 h, AUC_3–7_ is the area under the curve from 3 to 7 h, and AUC_7–12_ is the area under the curve from 7 to 12 h.
- Fed Study: log-transformed AUC_0–4_, AUC_4–8_, and AUC_8–12_, where AUC_0–4_ is the area under the plasma concentration vs. time curve from 0 to 4 h, AUC_4–8_ is the area under the curve from 4 to 8 h, and AUC_8–12_ is the area under the curve from 8 to 12 h.

The selected pAUCs have been identified as the most appropriate parameters for drug bioavailability evaluation, which are responsible for ensuring a quick onset and sustained maintenance of the clinical response throughout the drug effect duration.

To assess the bioequivalence, predefined acceptance criteria were applied to the 90% confidence interval for the ratio of the test and reference (T/R) formulations for the log-transformed data of Cmax and AUCs (AUC_0–t_, AUC_0–inf_, and pAUCs), where the acceptance range was set at 80.00–125.00% [11,12]. An analysis of variance (ANOVA) test was conducted to evaluate the effects of sequence, treatment, and period on these parameters.

### 2.7. Safety

All participants were continuously and carefully monitored in both the fasting and fed studies. Safety was assessed by monitoring baseline and ongoing vital signs including temperature, blood pressure, heart rate, and respiratory rate throughout the study. Additionally, laboratory tests (such as hematology, urinalysis, and blood biochemistry), physical examinations, and electrocardiograms (ECGs) were conducted at the beginning and conclusion of the study. Adverse events were assessed by the nursing and medical staff throughout the entire study. Subjects were instructed regarding the need to immediately report any undesirable symptoms or medical conditions during the study or after the hospitalization period. Adverse events were graded as mild, moderate, or severe, and their causality to the drug was determined by the medical staff as suspected or not suspected.

## 3. Results

### 3.1. Study Subjects

After the medical history assessment, verification of vital signs, physical examination, electrocardiogram, and routine laboratory tests, all subjects showed good health conditions and the absence of significant diseases. Table 1 shows the demographic subjects characteristics in both fasting and fed studies.

### 3.2. Sample Bioanalysis

The validated method covered all required tests, including the evaluation of the selectivity, concomitant medication interference, matrix effect, carry-over, calibration curve, precision, accuracy, reinjection reproducibility, and stabilities.

The method was linear in a concentration range of 25.0 to 30,000.0 pg/mL, and the lower limit of quantification (LLOQ) was 25.0 pg/mL. The method selectivity was demonstrated to be suitable by confirming that substances in the blank plasma samples did not affect MPH and IS retention times. In terms of precision and accuracy, the method was deemed appropriate for both within-assay (intra-run) and between-assay (inter-run) samples.

The stability assessments demonstrated that samples remained stable, with a variation less than 15% from the nominal value up to 6 h at room temperature (15 °C to 25 °C), and could remain stable for up to 52 h after extraction when stored in the auto-sampler at 10 °C. In terms of freeze–thaw stability, samples maintained their stability after undergoing four cycles of freezing in a standard freezer (−20 °C) and an ultrafreezer (−80 °C) followed by thawing at room temperature. Furthermore, the samples were proven to be stable and could be kept frozen for up to 168 days in an ultrafreezer (−80 °C). These stability tests are crucial to ensure proper sample storage prior to analysis, guaranteeing the accurate determination of drug concentrations.

The absence of carryover effects was confirmed since no predose samples from any participant showed the presence of MPH in plasma, confirming the appropriate washout period. Finally, all validation parameters met the predetermined acceptance criteria following ANVISA guidelines for bioanalytical method validation [19].

### 3.3. Pharmacokinetic Analysis

#### 3.3.1. Fasting Study

Ninety-six (96) healthy subjects were enrolled in the fasting study and eighty subjects (39 women and 41 men) completed the two study periods, being included in the pharmacokinetic and statistical analysis. A total of six subjects were withdrawn due to personal reasons, eight subjects were excluded due to adverse events, and two subjects were excluded due to drug abuse detection before the second drug administration period.

**Figure 1:**
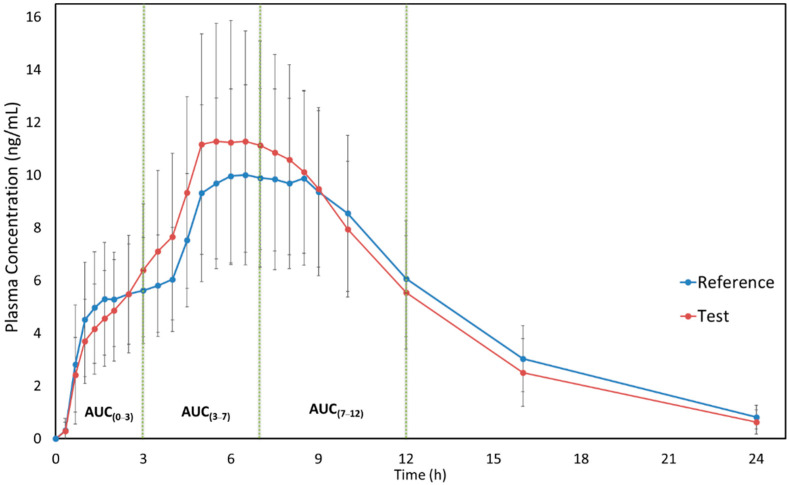
MPH plasma concentrations vs time curves after administration of test and reference formulations in healthy subjects under the fasting condition. Data are presented as mean and SD values (N = 80, healthy male and nonpregnant female subjects). The green dotted lines illustrate the intervals where the partial areas under the curve were calculated.

Figure 1 shows the mean plasma concentration versus time curves of MPH (reference and test formulations) when administered under fasting conditions. It is possible to observe the similarity of both test and reference pharmacokinetic profiles. Moreover, the sampling time can be considered adequate since it was possible to correctly describe the drug absorption and elimination phases. The pharmacokinetic parameters of MPH for both formulations are described in Table 2. The use of pAUC metrics was applied to ensure that, in fasting conditions, the products are therapeutic equivalents in different parts of the daily dosing interval.

#### 3.3.2. Fed Study

Fifty-two (52) healthy subjects were enrolled in the fed study, in which forty-six (24 women and 22 men) completed the two study periods and, therefore, were included in the pharmacokinetic and statistical analysis. A total of three subjects were withdrawn due to personal reasons, and three subjects were excluded due to adverse events before the second drug administration period.

**Figure 2:**
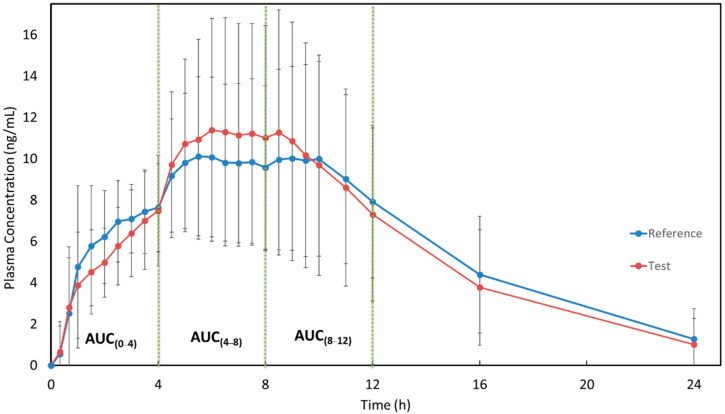
MPH plasma concentrations vs time curves after administration of test and reference formulations in healthy subjects under the fed condition. Data are present as mean and SD values (N = 46, healthy male and nonpregnant female subjects). The green dotted lines illustrate the intervals where the partial areas under the curve were calculated.

Figure 2 shows the mean plasma concentration versus time curves of MPH (reference and test formulations) when administered under fed conditions. It is possible to observe that the pharmacokinetic profiles of both test and reference formulations are very similar when administered under fed conditions. The pharmacokinetic parameters of MPH for both formulations are described in Table 3. The use of pAUC metrics was applied to ensure that, in the fed condition, the products are therapeutic equivalents in different parts of the daily dosing interval.

### 3.4. Bioequivalence Assessment

Table 4 and Table 5 present the test/reference geometric mean ratio for pharmacokinetic parameters Cmax, AUC_0–t_, AUC_0–inf_, and pAUCs, and the 90% CIs for the bioequivalence analysis for the fasting and fed studies, respectively.

Regarding the fasting study, the result obtained for Cmax is very similar to those obtained for AUC_3–7_. This happened mainly because the mean values of Tmax were between 3 and 7 h, and the median was estimated at 6.50 h for both formulations. The ratios were shown to be displaced upwards (above 112%); the CV_ws_ were considered to be low, presenting a result below 16%; and the power estimates (TOST method) were above 94%. For AUC_0–3_ the geometric mean ratio was around 94%, and the power estimate was higher than 99%, with similar low CV_ws_ values. The results obtained for AUC_7–12_, AUC_0–t_, and AUC_0–inf_ were also similar presenting ratios centered on the CI, CV_ws_ ranging between 8 and 11%, and all power estimates reaching 100%.

Concerning the fed study, the geometric mean ratios for Cmax and AUC_0–4_ showed a slight shift, presenting values around 111% and 88%, respectively. The CV_ws_ for both parameters could be considered low, however, the power (TOST method) estimates did not reach 80%. So, the CV_ws_ equality test was performed, and the results indicate that both AUC_0–4_ and Cmax present variability equivalence in terms of variance. For AUC_4–8_, despite the observed geometric mean ratio having been slightly displaced upwards, the power estimate was higher than 93%. For AUC_8–12_, AUC_0–t_, and AUC_0–inf_ the results were very similar. The ratios presented values around 96 and 99%, estimated CV_ws_ lower than 17%, and power estimates that reached 100%.

The ANOVA *p*-values did not demonstrate statistically relevant differences for all evaluated parameters for sequence and period fixed effects at 10% and 5% significance levels, respectively. All 90% CIs of test/reference geometric mean ratios for both studies (fasting and fed) fell within the bioequivalence acceptance range of 80.00–125.00%, established by ANVISA [11,12]. So, the two MPH extended-release formulations (test and reference) are bioequivalent in terms of the rate and extent of absorption.

In addition to the bioequivalence data analysis, the present study did not show significant differences (*p* < 0.05) in the pharmacokinetic parameters regarding subject gender. Table 6 summarizes the obtained pharmacokinetic data for both test and reference formulations, according to gender for the fasting and fed studies, respectively. Our results agree with the statement in the Concerta^®^ label that, in healthy adults, the mean dose-adjusted AUC_(0–inf)_ data were 36.7 ng/mL.h in men and 37.1 ng/mL.h in women, with no significant differences between the two groups [6].

### 3.5. Safety

In the fasting study, a total of 90 adverse events were reported by 53 of the 96 participants for both test and reference formulations; the most common were headache (30.0%) and leukocyturia (17.8%) (Table 7). In turn, in the fed study a total of 55 adverse events were reported by 30 of the 52 participants for both test and reference formulations. The most common adverse events were leukocyturia (18.2%) and headache (14.5%) (Table 8). Regarding the fasting study, eight adverse events were classified as causality suspected related to the drug and twelve of them were considered causality not suspected to the drug, whilst in the fed study five adverse events were classified as causality suspected related to the drug and thirteen of them were considered causality not suspected to the drug.

## 4. Discussion

The development of generic products for multiphasic release formulations is a challenge, mainly in terms of galenic and clinical studies. In this work, we have studied two formulations of MPH multiphasic release tablets (test and reference) in order to assess the bioequivalence for generic product registration. Both test (Consiv^®^) and reference (Concerta^®^) formulations demonstrated an ascending pharmacokinetic profile with plateau concentrations around 4 to 6 h; a similar behavior was observed by Markowitz and collaborators (2003) for Concerta^®^ [20].

Schapperer and collaborators (2014) [21] performed bioequivalence studies comparing Concerta^®^ with an MPH osmotic-controlled release (OCR) tablet and presented data for fasting (N = 24) and fed (N = 21) conditions [17]. For the MPH OCR (54 mg) in the fasting study, a Cmax of 10.89 ± 2.09 ng/mL, AUC_0–t_ of 116.70 ± 26.52 ng.h/mL, AUC_0–inf_ of 121.98 ± 28.07 ng.h/mL, Tmax of 5.81 ± 1.01 h, and t_1/2_ of 4.36 ± 0.55 h were found. On the other hand, for Concerta^®^ (54 mg) a Cmax of 12.11 ± 2.95 ng/mL, AUC_0–t_ of 121.43 ± 27.00 ng.h/mL, AUC_0–inf_ of 125.36 ± 28.12 ng.h/mL, Tmax of 6.96 ± 1.56 h, and t_1/2_ of 3.88 ± 0.46 h were found. In the fed study for the MPH OCR (54 mg), a Cmax of 12.55 ± 3.36 ng/mL, AUC_0–t_ of 141.02 ± 43.11 ng.h/mL, AUC_0–inf_ of 148.92 ± 47.46 ng.h/mL, Tmax of 5.17 ± 0.64 h, and t_1/2_ of 4.52 ± 0.92 h were found. Moreover, for Concerta^®^ (54 mg) a Cmax of 13.35 ± 4.04 ng/mL, AUC_0–t_ of 148.57 ± 47.73 ng.h/mL, AUC_0–inf_ of 154.38 ± 51.61 ng.h/mL, Tmax of 8.19 ± 2.64 h, and t_1/2_ of 3.79 ± 0.52 h were found. The pharmacokinetic parameters shown in the work by Schapperer and collaborators (2014) [21] are similar to our results (Table 2 and Table 3). Unfortunately, it was not possible to compare the pAUC metric values because the authors calculated different intervals for the fasting study and did not present these metrics for the fed study.

A comparative bioavailability study was performed by Reiz and collaborators (2008) [22] evaluating a multilayer-based bead and an osmotic system formulation [18]. The study was conducted with twenty-one healthy subjects, the formulations were administered under fed conditions, and the authors evaluated the pAUC metrics using 20 mg of MPH. Despite the differences in pharmacokinetic parameters caused by the different drug doses, the author’s plasma concentration versus time profile was very similar to our results. Notably, the formulations were designed to provide a multiphasic behavior, including a rapid initial release and a second sustained one.

Considering the pharmacokinetic data described in the Concerta^®^ label [6], MPH concentrations increase rapidly reaching an initial maximum at about 1 h, followed by gradual ascending concentrations over the next 5 to 9 h after which a gradual decrease begins. Similar behavior was presented in our study (Figure 1 and Figure 2) for both formulations. Additionally, the Concerta^®^ label describes that Tmax occurs between 6 to 10 h and T_1/2_ of 3.5 h. In our fasting study, Tmax was 6.44 (±1.33) h for the test formulation and 6.97 (±1.23) h for the reference formulation. For the fed study, we found a Tmax of 6.35 (±2.35) h for the test formulation and 6.44 (±2.59) h for the reference formulation. Regarding the T_1/2_, our results (3.6–4.6 h) were similar to those described in the Concerta^®^ label [6]. These comparisons are important to guarantee that the study design, sample size, and blood sampling adopted in our work were adequate to correctly describe the pharmacokinetic profile of each formulation and, consequently, to assess the bioequivalence. Moreover, it is important to mention that the adopted study design was adequate to calculate pAUC metrics (following the FDA requirements [14]) and necessary measures to compare the formulations in different parts of the daily dosing interval.

This study compared the bioavailability of test and reference MPH 54 mg extended-release tablets in different intervals, and also monitored safety and tolerability under fasting and fed conditions. Here, we provide important information about pAUC metrics that could help the understanding of the MPH PK/PD relationship, and the oral intake of test or reference MPH tablets showed similar favorable safety profiles. However, it is important to exercise caution when evaluating adverse events resulting from a bioequivalence study, as this was conducted with healthy subjects and the drug was administered as a single dose. Continuous pharmacovigilance and a post-marketing surveillance plan are responsible for any safety concern monitoring.

## 5. Conclusions

Based on 90% CI of test/reference geometric mean ratios for Cmax, AUC_0–t_, AUC_0–inf_, AUC_0–3_, AUC_3–7_, and AUC_7–12_ in the fasting study; and Cmax, AUC_0–t_, AUC_0–inf_, AUC_0–4_, AUC_4–8_, and AUC_8–12_ in the fed study, the two MPH extended-release formulations can be considered to be bioequivalent. According to the obtained data, it can be confirmed that drug exposure is equivalent between test and reference formulations when administered under both conditions (fasting and fed). Both formulations were well tolerated and no relevant differences in safety profiles between them were found. In conclusion, considering that the bioavailability of the test and reference formulations are essentially similar as well as the safety data observed in these studies, the branded-generic formulation registered in Brazil by Adium S.A., Consiv^®^ is considered to be bioequivalent to the reference formulation (Concerta^®^) and it is expected to produce the same therapeutic response. Thus, these products may be considered interchangeable in medical practice.

## Figures and Tables

**Table 1:** Demographic characteristics in fasting and fed studies.

	Fasting (N = 80)	Fed (N = 46)
Age (years)		
Mean _(±SD)_	31.79 _(±7.57)_	31.85 _(±7.47)_
Median	31.50	32.00
Range	18–49	18–46
Weight (kg)		
Mean _(±SD)_	67.6 _(±8.76)_	70.3 _(±8.48)_
Median	66.18	69.13
Range	52.0–95.0	50.2–93.0
Height (m)		
Mean _(±SD)_	1.67 _(±0.09)_	1.69 _(±0.09)_
Median	1.66	1.69
Range	1.47–1.89	1.52–1.85
BMI (kg/m^2^)		
Mean _(±SD)_	24.14 _(±2.50)_	24.77 _(±2.47)_
Median	24.14	25.15
Range	19.23–28.57	19.99–28.58
Sex (n [%])		
Male	44 (55%)	24 (52.2%)
Female	36 (45%)	22 (47.8%)

**Table 2:** Pharmacokinetic parameters of MPH extended-release tablets administered under fasting conditions in healthy subjects (N = 80). Data expressed as mean _(±SD)_.

Parameter	Test	Reference
Cmax (ng/mL)	12.36 _(±4.35)_	10.94 _(±3.61)_
AUC_0–t_ (ng/mL·h)	119.34 _(±39.34)_	119.10 _(±40.54)_
AUC_0–inf_ (ng/mL·h)	122.96 _(±41.75)_	124.23 _(±42.83)_
AUC_0–3_ (ng/mL·h)	11.20 _(±4.10)_	12.02 _(±4.33)_
AUC_3–7_ (ng/mL·h)	38.18 _(±13.90)_	32.49 _(±10.62)_
AUC_7–12_ (ng/mL·h)	42.21 _(±13.03)_	42.01 _(±14.41)_
Tmax (h)	6.44 _(±1.33)_	6.97 _(±1.23)_
T_1/2_ (h)	3.67 _(±0.62)_	4.07 _(±0.62)_
kel (1/h)	0.194 _(±0.033)_	0.174 _(±0.027)_

Cmax, maximum plasma concentration; Tmax, time to reach Cmax; AUC_0–t_, area under the concentration–time curve from zero to 24 h; AUC_0–3_, area under the concentration–time curve from zero to 3 h; AUC_3–7_, area under the concentration–time curve from 3 to 7 h; AUC_7–12_, area under the concentration–time curve from 7 to 12 h; AUC_0–inf_, area under the concentration–time curve extrapolated to infinity; t_1/2_, elimination half-life; kel, elimination constant.

**Table 3:** Pharmacokinetic parameters of MPH extended-release tablets administered under fed conditions in healthy subjects (N = 46). Data expressed as mean _(±SD)_.

Parameter	Test	Reference
Cmax (ng/mL)	13.78 _(±6.12)_	12.00 _(±4.49)_
AUC_0–t_ (ng/mL·h)	141.52 _(±61.65)_	145.41 _(±60.08)_
AUC_0–inf_ (ng/mL·h)	149.34 _(±72.33)_	155.1186 _(±76.89)_
AUC_0–4_ (ng/mL·h)	18.98 _(±5.62)_	21.68 _(±6.54)_
AUC_4–8_ (ng/mL·h)	42.85 _(±17.88)_	38.62 _(±13.55)_
AUC_8–12_ (ng/mL·h)	38.43 _(±19.85)_	37.82 _(±16.62)_
Tmax (h)	6.35 _(±2.35)_	6.45 _(±2.59)_
T_1/2_ (h)	4.13 _(±2.11)_	4.37 _(±0.98)_
kel (1/h)	0.185 _(±0.043)_	0.165 _(±0.030)_

Cmax, maximum plasma concentration; Tmax, time to reach Cmax; AUC_0–t_, area under the concentration–time curve from zero to 24 h; AUC_0–4_, area under the concentration–time curve from zero to 4 h; AUC_4–8_, area under the concentration–time curve from 4 to 8 h; AUC_8–12_, area under the concentration–time curve from 8 to 12 h; AUC_0–inf_, area under the concentration–time curve extrapolated to infinity; t_1/2_, elimination half-life; kel, elimination constant.

**Table 4:** Geometric mean ratio, confidence intervals (90%), and CV_ws_ for fasting study (N = 80).

Parameter *	Geometric Mean Ratio (%)	90% CI	CV_ws_
Cmax	112.91	108.52–117.47	15.13
AUC_0–t_	100.81	98.57–103.11	8.59
AUC_0–3_	93.55	89.17–98.14	18.35
AUC_3–7_	116.73	112.76–120.83	13.20
AUC_7–12_	101.59	98.79–104.47	10.64

* Parameters logarithmically transformed.

**Table 5:** Geometric mean ratio, confidence intervals (90%), and CV_ws_ for fed study (N = 46).

Parameter *	Geometric Mean Ratio (%)	90% CI	CV_ws_
Cmax	110.84	102.22–120.19	23.40
AUC_0–t_	97.03	93.80–100.38	9.69
AUC_0–4_	87.57	82.29–93.19	17.88
AUC_4–8_	108.56	100.79–116.94	21.43
AUC_8–12_	99.11	93.62–104.91	16.35

* Parameters logarithmically transformed.

**Table 6:** Pharmacokinetic parameters of MPH extended-release tablets administered under fasting and fed conditions in men and women. Data expressed as mean _(±SD)_.

Fasting Study			Fed Study		
Parameter	Men (N = 44)	Women (N = 36)	Parameter	Men (N = 24)	Women (N = 22)
Cmax (ng/mL)	11.87 _(±4.22)_	9.82 _(±2.29)_	Cmax (ng/mL)	12.18 _(±5.55)_	11.81 _(±3.06)_
AUC_0–t_ (ng/mL·h)	129.49 _(±45.89)_	106.39 _(±28.59)_	AUC_0–t_ (ng/mL·h)	152.07 _(±73.98)_	138.15 _(±40.42)_
AUC_0–inf_ (ng/mL·h)	135.63 _(±48.81)_	110.30 _(±29.18)_	AUC_0–inf_ (ng/mL·h)	165.64 _(±98.32)_	143.64 _(±42.61)_
AUC_0–3_ (ng/mL·h)	12.29 _(±4.83)_	11.69 _(±3.67)_	AUC_0–4_ (ng/mL·h)	21.89 _(±7.69)_	21.46 _(±5.16)_
AUC_3–7_ (ng/mL·h)	34.60 _(±12.37)_	29.91 _(±7.38)_	AUC_4–8_ (ng/mL·h)	39.42 _(±15.85)_	37.74 _(±10.79)_
AUC_7–12_ (ng/mL·h)	45.90 _(±16.21)_	37.26 _(±10.19)_	AUC_8–12_ (ng/mL·h)	39.39 _(±20.56)_	36.10 _(±11.09)_

**Table 7:** Adverse events reported during hospitalization in fasting study.

Adverse Event	Prevalence %	Causality	Intensity
Headache	30.0%	Not suspect	Mild
Leukocyturia	17.8%	Not suspect	Mild
Nausea	12.2%	Not suspect	Mild
Hematuria	5.6%	Not suspect	Mild
Hypertension	4.4%	Suspect	Mild
Dizziness	4.4%	Not suspect	Mild
Vomiting	3.3%	Not Suspect	Mild
Tachycardia	3.3%	Suspect	Mild
Hyperuricemia	3.3%	Not suspect	Mild
Odynophagia	2.2%	Suspect	Mild
AST increase	2.2%	Suspect	Mild
Hypercholesterolemia	2.2%	Not suspect	Mild
Weakness	1.1%	Suspect	Mild
Malaise	1.1%	Not suspect	Mild
Hyperbilirubinemia	1.1%	Suspect	Mild
ALT Increase	1.1%	Suspect	Mild
Thrombocytopenia	1.1%	Not Suspect	Mild
Swelling	1.1%	Suspect	Mild
Epigastric Pain	1.1%	Not Suspect	Mild
Menstrual Cramps	1.1%	Not Suspect	Mild

ALT: Alanine transaminase; AST: Aspartate transaminase.

**Table 8:** Adverse events reported during hospitalization in the fed study.

Adverse Event	Prevalence %	Casualty	Intensity
Leukocyturia	18.2%	Not Suspect	Mild
Hyperbilirubinemia	14.5%	Suspect	Mild
Headache	14.5%	Not Suspect	Mild
Hypertriglyceridemia	12.7%	Not Suspect	Mild
Hematuria	5.5%	Not Suspect	Mild
Hypercholesterolemia	5.5%	Not Suspect	Mild
AST Increase	3.6%	Suspect	Mild
Nausea	3.6%	Not Suspect	Mild
Abdominal Distress	3.6%	Not Suspect	Mild
Hypertension	3.6%	Suspect	Mild
Glycosuria	1.8%	Not Suspect	Mild
Decreased Serum Segmented Neutrophils	1.8%	Not Suspect	Mild
Uric Acid Increase	1.8%	Not Suspect	Mild
Increased Serum Segmented Neutrophils	1.8%	Not Suspect	Mild
Dizziness	1.8%	Not Suspect	Mild
Tonsillitis	1.8%	Not Suspect	Mild
Confusional State	1.8%	Suspect	Mild
Palpitation	1.8%	Suspect	Mild

AST: Aspartate transaminase

## Data Availability

The data are not publicly available due to confidentiality reasons.
